# Synthesis and Pharmacological Valorization of Derivatives of 4-Phenyl-1,5-Benzodiazepin-2-One

**DOI:** 10.1155/2018/6042602

**Published:** 2018-04-01

**Authors:** Terence Nguema Ongone, Redouane Achour, Mostafa El Ghoul, Latifa El Ouasif, Khalid Taghzouti, Meryem El Jemli, Yahia Cherrah, Katim Alaoui, Amina Zellou

**Affiliations:** ^1^Laboratory of Heterocyclic Organic Chemistry, Drug Sciences Research Center, URAC 21, Pole of Competence Pharmacochemistry, Faculty of Sciences, Mohammed V University, Avenue Ibn Battouta, BP 1014, Rabat, Morocco; ^2^PharmacodynamyResearch Team PRT, Laboratory of Pharmacology and Toxicology, Faculty of Medicine and Pharmacy, Mohammed V University, BP 6203, Rabat, Morocco; ^3^Laboratory of Animal Physiology, Department of Biology, Faculty of Science, Mohammed V University, Avenue Ibn Battouta, BP 1014, Rabat, Morocco

## Abstract

The objective of our work is to make a pharmacological study of molecules derived from 4-phenyl-1,5-benzodiazepin-2-one carrying long chains so that they have a structure similar to surfactants, with the benzodiazepine as a hydrophilic head and a carbon chain as a hydrophobic tail. First, we studied the acute toxicity of the above mentioned 4-phenyl-1,5-benzodiazepin-2-one derivatives. This study was conducted according to OECD 423 guidelines in female mice and revealed that these compounds are nontoxic. We then assessed the psychotropic effects of our products on the central nervous system (CNS). The results obtained show that 4-phenyl-1,5-benzodiazepin-2-one has no sedative effect at therapeutic doses of 100 and 200 mg/kg. On the other hand, its long-chain derivatives possess them. Moreover, all these products have no cataleptic and hypnotic effects at the doses studied. But at 100 mg/kg, these compounds all have the ability to significantly prolong the hypnotic effect of thiopental sodium.

## 1. Introduction

The development of heterocyclic organic chemistry has been very important for humans. Indeed, this part of the chemistry allows the synthesis of bioactive molecules used in the pharmaceutical industry for the preparation of drugs. In this family, benzodiazepines have been shown to be pharmacologically important since they exert anxiolytic, analgesic, anticonvulsant, antidepressant, cataleptic, hypnotic, myorelaxant, and sedative [1–3] effects on the central nervous system (CNS). This is the case of 7-bromo-5-(2-pyridinyl)-1,4-benzodiazepin-2-one (bromazepam) marketed for its strong anxiolytic effect and its effective hypnotic effect [4] and 7-chloro-1-(cyclopropylmethyl)-5-phenyl-1,3-dihydro-2H-1,4-benzodiazepin-2-one also known as “prazepam” marketed especially for its anxiolytic effect [5]. Other studies report that benzodiazepines have anti-inflammatory, antiviral, anti-HIV, antimicrobial, and antitumor activities [6].

**Figure 1:**
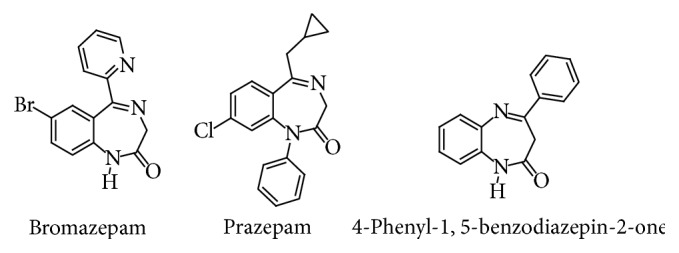
Structures of bromazepam, prazepam, and 4-phenyl-1,5-benzodiazepin-2-one.

Given the pharmacological importance of benzodiazepines, we set ourselves the objective of the pharmacological study of 4-phenyl-1,5-benzodiazepin-2-one (Figure 1) and its derivatives in such a way that the latter have a structure similar to surfactants, with the benzodiazepine as a hydrophilic head and a carbon chain as a hydrophobic tail. Indeed, the literature reports a large number of works showing that, in the pharmaceutical field, the surfactants can carry hydrophilic and hydrophobic active ingredients of the drugs into the cells; they can also protect them and significantly reduce their toxicity [7].

## 2. Materials and Methods

### 2.1. Chemistry

#### 2.1.1. Choice of Products to Synthesize

Our objective is the pharmacological study of derivatives of 4-phenyl-1,5-benzodiazepin-2-one with long chains **5a–d**. To prepare them, we used the reagents that were available to us, namely, 1-bromooctane, 1-bromononane, 1-bromodecane, and 1-bromododecane.

#### 2.1.2. Synthesis of 4-Phenyl-1,5-Benzodiazepin-2-One and Its Derivatives

**Scheme 1:**
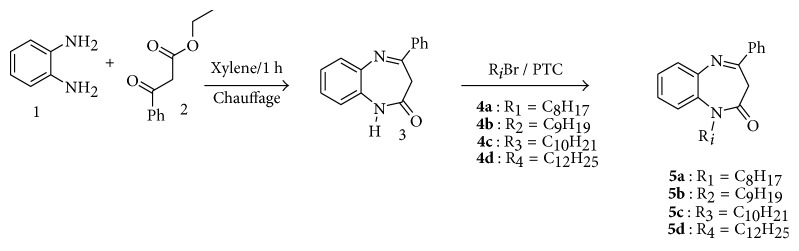


We prepared 4-phenyl-1,5-benzodiazepin-2-one **3** by condensation of 1,2-phenylenediamine 1 with ethyl benzoyl acetate 2 at xylene reflux for one hour [8]. This compound was subjected to a series of alkylation reactions, under the conditions of phase-transfer catalysis (PTC), with 1-bromooctane, 1-bromononane, 1-bromodecane, and 1-bromododecane to obtain 1-octyl(1-nonyl, 1-decyl, 1-dodecyl)-4-phenyl-1,5-benzodiazepin-2-one (Scheme 1).

The structures of these compounds were confirmed by spectroscopic analyses (1H NMR and 13C NMR) and mass spectrometry. The nuclear magnetic resonance spectra (1H, 13C) were recorded on an AVANCE 300 Bruker device operating at 300 MHz, in solution in deuterated chloroform. The chemical shifts are given in ppm relative to the TMS internal reference, and the mass spectra were made by electronic impact using the VARIAN MAT 311A. Moreover, these products are obtained according to the following operating modes.

4-Phenyl-1,5-benzodiazepin-2-one **3**: 0.01 mol of 1,2-phenylenediamine and 0.011 mol of ethyl benzoylacetate are poured into a 250 ml flask containing 50 ml of xylene, and the whole is left to reflux for 1 h. After the reaction and during cooling, a precipitate forms which is filtered and washed with ethanol and dried to obtain a yellowish-colored powder. This product is obtained with a yield of 75% (mp 196–198°C/EtOH).

1-alkyl-4-phenyl-1,5-benzodiazepin-2-one **5a-d:** they are obtained by reacting, in a 100 ml flask containing 30 ml of tetrahydrofuran, 0.0021 mol of 4-phenyl-1,5-benzodiazepin-2-one and 0.00315 mol of 1-bromoalkane in the presence of 0.0042 mol of carbonate. of potassium (weak base) and a pinch of tetrabutylammonium bromide (TBAB). The reaction is stirred at room temperature for 24 hours. The 1-alkyl-4-phenyl-1,5-benzodiazepin-2-one thus prepared is purified by chromatography on silica gel with hexane/ethyl acetate eluent (80/20) in a yield of 83%. The spectral data for these compounds are summarized in Table 1.

#### 2.1.3. Solubility of Products **5a–d**

The products **5a-b** are soluble in several solvents such as ethanol, methanol, dimethylformamide, and tetrahydrofuran.

### 2.2. Evaluation of Acute Toxicity

#### 2.2.1. Animals

We used the Swiss mice (20–30 g) for our work. These animals were raised in the Laboratory of Pharmacology and Toxicology of the Faculty of Medicine and Pharmacy of Rabat. All animals were housed in collective cages at controlled temperature (25°C ± 2°C), relative humidity (40 and 70%), and artificially lit chambers on a cycle of 12 h of light/12 h of darkness with the free access to water and standard power. The use of the animals has been done in accordance with the Laboratory Animal Use Guidelines [9, 10].

#### 2.2.2. Method Used

The acute toxicity study of our products was conducted according to the OECD (Organization for Economic Co-operation and Development) Guidelines 423 [11]. The Swiss, female, and healthy mice are fasted for 12 hours before experiments with ad libitum water. The animals were randomly divided into seventeen groups (*n*=6). The first group (control group) received gum Arabic VO (1%) (vehicle indicator). The other groups are each treated with one of the products tested at the dose of 300 and 2000 mg/kg. For each dose, the test is performed twice to be sure of the result obtained for each of the products studied.

Mice were observed for general behavioral symptoms, changes in body weight, dangerous symptoms, and mortality during the first 4 hours and then for 14 days after administration of the products. The 50% lethal dose (LD50) was estimated according to the method described in the OECD Guidelines 423.

### 2.3. Psychotropic Activities

#### 2.3.1. Catatonigenic Action

An animal will be considered as cataleptic if it is allowed to cross the anterior and later legacy homolateral. Note the time this activity takes place if it occurs. It should be noted that the cataleptic state is deeper than the hypnotic state [12, 13]. The animals were administered oral therapeutic doses of 100 and 200 mg/kg of the vehicle and drug.

#### 2.3.2. Hypnotic Action

The hypnotic action of a substance is its capacity to give sleep to the subject to which it is administered. Animals (mice or rats) that have received a therapeutic dose of LD50 of a substance will be considered hypnotized if they lose their straightening reflexes when put on their backs.

For this study, the mice were orally administered at doses of 100 and 200 mg/kg. Two parameters are noted: the time of falling asleep (TE), which is the time elapsing between the administration of the substance and the suppression of the righting reflex, and the sleep time (TS), which is the time elapsing between the disappearance of the reflex correction and its reappearance [12, 14].

#### 2.3.3. Sedative Activity

The sedative activity of benzodiazepines, that is, their ability to reduce alertness and psychomotor reactivity in an individual, is detected in mice through a series of behavioral tests: the tensile test, the test of chimney, the drill plate test, and the Rotarod test, in comparison with a reference substance.

For our study, we will study the sedative effect of 4-phenyl-1,5-benzodiazepin-2-one and its derivatives **5a–d** at therapeutic doses of 100 and 200 mg/kg (vo), in comparison with bromazepam at the therapeutic dose of 20 mg/kg (vo). The administration of the products to the animals is done thirty minutes before each test, and each animal is individually tested in one of the tests studied.

- Traction test

Placing the forepaws of the mice in a small twisted wire (1 mm diameter and 15 cm long) rigidly supported above the bench top did the screening of animals.

Normal mice grasped the wire with forepaws, and when allowed to hang free, they placed at least one hind foot on the wire within 5 seconds. Inability to put up at least one hind foot considered failure in the traction test; also, the behaviors of animals were recorded during this experiment [15, 16].

(ii) Chimney test

Chimney test of Boissier 1961 was used where each mouse was introduced into the vertical glass tube 30 cm in length and 28 mm in diameter with the head forward. As soon as the mouse reaches the bottom of the tube on all fours, she tries to put it back up.

The time required for the mouse to climb backwards out of the cylinder was noted. Cutoff time was 240 sec. A normal mouse typically attempts to escape in thirty seconds, and the mice are considered as subject to the sedative effect when performing the rise of cylinder greater than 30 seconds [17, 18].

(iii) Hole-board test

The board is 40 cm × 40 cm and 2.2 cm thick. It has 16 holes of 3 cm diameter, and it is made of grey Perspex. The matt finishing of the upper panel avoids reflections which may alter the animal behavior. The mice were placed in the center of the hole board and allowed to freely explore the apparatus for 5 minutes. The numbers of head pokes and the time of dipping during a 5-minute period were recorded [19].

(iv) Rota-Rod test

In this test, mice were selected 24 h prior to the test by choosing only those that were able to remain successfully on the revolving bar (14 rpm) of the Rota-Rod apparatus (Ugo Basile, model 7600) for two consecutive periods of 60 seconds. Motor performance was evaluated at 30, 60, and 120 minutes following treatments, and the amount of time of permanence (s) on the revolving bar during a 60-second period was recorded [20].

#### 2.3.4. Drug Interaction

This study consists of combining two substances administered simultaneously to the animals, one of which is pharmaceutically known for its hypnotizing effect, while the other is the product that it is desired to test. In some cases, there may be an increase in the activity of the drug for the same dose. In others, a reduction or even an inhibition of the effectiveness of the treatment may occur. In the first case, we speak of synergistic interaction and, in the second, of antagonistic interaction, but we can also have potentiation. For this test as for the hypnotic test, the time of falling asleep (TE) and the sleeping time (TS) are measured.

We carried out a drug interaction study of our products with thiopental sodium in order to check their influence (at 100 mg/kg) on the hypnotic action of the latter 40 mg/kg in mice, compared with l bromazepam interaction (30 mg/kg) - thiopental sodium (40 mg/kg). Three lots of 5 animals will be used:

- The first batch will receive thiopental sodium at 40 mg/kg: this is the control batch.
- The second batch will simultaneously receive thiopental sodium 40 mg/kg + bromazepam 30 mg/kg: this is the reference lot; the third batch will receive the product mixture **5a–d** 100 mg/kg + 40 mg/kg of thiopental sodium: this is the batch of the product tested.

## 3. Results

### 3.1. Acute Toxicity

#### 3.1.1. Acute Toxicity of 4-Phenyl-1,5-Benzodiazepin-2-One

Kanyonga et al. [3] conducted a study of the acute toxicity of 4-phenyl-1,5-benzodiazepin-2-one. They showed that the lethal dose 50% (LD50) is 1617.08 mg/kg.

#### 3.1.2. Acute Toxicity of 4-Phenyl-1,5-Benzodiazepin-2-One Derivatives

After administration of each dose, we found that it was possible for us to have a negative effect on the effects of these diseases.

Furthermore, follow-up of animals during the days of administration and administration of the animal at doses of 300 and 2000 mg/kg and their weights remain relatively stable. This result shows that the LD50 of products is greater than 2000 mg/kg and belongs to category V in the global chemical classification system. These compounds do not cause obesity or anorexia.

### 3.2. Catatonigenic Action

Our study shows that 4-phenyl-1,5-benzodiazepin-2-one and its derivatives do not have catatonigenic (cataleptic) effects on the central nervous system at therapeutic doses of 100 and 200 mg/kg.

### 3.3. Hypnotic Action

Our study reveals that 4-phenyl-1,5-benzodiazepin-2-one and its derivatives do not have hypnotic effects on the central nervous system at therapeutic doses of 100 and 200 mg/kg.

### 3.4. Sedative Activity

#### 3.4.1. Sedative Activity of 4-Phenyl-1,5-Benzodiazepin-2-One

- Traction test

The average recovery time evaluated 30 min after administration of the product to the animals is slightly higher than that of the control group, which reflects a high level of vigilance in treated animals. In addition, this time decreases when one passes from the dose of 100 to 200 mg/kg (Table 2), and no case of fall was recorded during this test.

(ii) Test of the chimney

For this test, the animals tested with 4-phenyl-1,5-benzodiazepin-2-one at doses of 100 and 200 mg/kg show no sedative effect, as they only take less than 10 seconds to recover. The test piece is shown in Table 2. This means that they retain their sense of initiative.

(iii) Hole-board test

4-Phenyl-1,5-benzodiazepin-2-one slightly decreases the cumulative number of holes explored by animals (in relation to curiosity) and the number of spaces traveled between two holes (in relation to motor activity), in comparison with the control group and the reference substance (Table 2). This result reflects the absence of a sedative effect at therapeutic doses of 100 and 200 mg/kg.

(iv) Rota Rod test

In this test, we observe that the animals put more than 120 seconds on the rotating stem before falling when we give them our product at therapeutic doses of 100 and 200 mg/kg (Table 2). This result reveals that the level of vigilance of the latter remains very high. Hence, there is absence of a sedative effect for this compound at these doses.

#### 3.4.2. Sedative Activity of 4-Phenyl-1,5-Benzodiazepin-2-One Derivatives

- Traction test

The results of this test reveal that products **5a–d** reduce the vigilance of the animals to which they are administered, since they have difficulty straightening up on the wire (Table 2). Indeed, during the experiment, we have recorded rates of falls ranging from 8 to 12% for products **5a** and **5b** at the therapeutic doses studied.

(ii) Chimney test

The results of this test reveal a loss of curiosity in animals receiving doses of **5a–d** products, as they do not rapidly show the desire to leave the test tube as do the control animals (Table 3).

(iii) Hole-board test

The results of the hole-board test support those of the chimney test. Indeed, this test shows that the exploratory curiosity of the animals decreases significantly when treated with the compounds **5a–d**, compared with the control animals (Table 3).

(iv) Rota Rod Test

The results of this test confirm the loss of vigilance observed during the traction test. Indeed, the animals subjected to the products **5a-b** lose their capacity to remain on the rotating rod by staying there for less than one minute, compared with the controls which remain there for more than 2 minutes (Table 3).

### 3.5. Drug Interaction

#### 3.5.1. Drug Interaction with 4-Phenyl-1,5-Benzodiazepin-2-One

4-Phenyl-1,5-benzodiazepin-2-one has no cataleptic effect or hypnotic effect at the therapeutic dose of 100 mg/kg as previously shown. On the other hand, when administered concomitantly with thiopental sodium, it prolongs very significantly the hypnotic effect of the latter (Table 4).

#### 3.5.2. Drug Interaction with 4-Phenyl-1,5-Benzodiazepin-2-One Derivatives

The results of this study show that, at the therapeutic dose 100 mg/kg, all the products tested significantly increase the sleep time in the mice used (Table 5).

## 4. Discussion

In this work, we have shown that the addition of alkyl chains to 4-phenyl-1,5-benzodiazepin-2-one significantly decreases the acute toxicity of the latter. Indeed, the acute toxicity study conducted by Kanyonga et al. [3] on this compound reveals that its LD50 is 1617.08 mg/kg, whereas our study on its long-chain derivatives **5a–d** shows that their respective LD50s are above 2000 mg/kg. This result is in line with the work of Soussan et al. [21].

Furthermore, we have shown that 4-phenyl-1,5-benzodiazepin-2-one does not have catatonigenic and hypnotic effects on the central nervous system (CNS) at therapeutic doses of 100 and 200 mg/kg. However, it has a very low sedative effect at these doses, compared with bromazepam 20 mg/kg (Table 2), and has the ability to prolong the hypnotic effect of thiopental sodium (Table 4). These results are in agreement with those obtained by Kanyonga et al. [22] on other derivatives of this molecule, which show that benzodiazepines have a good ability to prolong the hypnotic effect of known substances. All of this work reveals the pharmacological importance of benzodiazepines and justifies the fact that a large number of researchers have embarked on the synthesis of new benzodiazepines that may have interesting biological activities [23]. However, these results are still insufficient to consider the use of 4-phenyl-1,5-benzodiazepin-2-one and its short-chain derivatives as active ingredients of possible drugs at doses studied. Therefore, in order to improve the pharmacological activities of 4-phenyl-1,5-benzodiazepin-2-one, we introduced long alkyl chains at the nitrogen atom in position 1 to obtain **5a–d** products whose structures are similar to surfactants. The results of the study of the psychotropic activity obtained with these various compounds allow us to observe that the alkyl chains introduced do not improve the catatonigenic and hypnotic effects of 4-phenyl-1,5-benzodiazepin-2-one with therapeutic doses studied but slightly increase sedative activity at the same doses (Tables 2 and 3). These results are in agreement with those of the study conducted by Minnih et al. [24] on the molecules of the same family. In addition, we find that the sedative activity of **5a–d** molecules increases when we go from the 100 mg/kg dose to the 200 mg/kg dose, and it decreases as the size of the added alkyl chain increases. This result is consistent with the structure-activity relationship (Table 3). The difference in results observed between 4-phenyl-1,5-benzodiazepin-2-one and its derivatives could be explained by the fact of having introduced long alkyl chains which would allow them easy access to the GABA receptor. In addition, we simultaneously administered 40 mg/kg of thiopental sodium and 100 mg/kg of each of the **5a–d** products to the animals to study the drug interaction. We had a mean sleep time of 153 minutes for **5a**, 118 minutes for **5b**, 88 minutes for **5c,** and 82 minutes for **5d**. These results reveal that, like 4-phenyl-1,5-benzodiazepin-2-one, its long-chain carbon derivatives have the ability to satisfactorily increase the hypnotic effect of thiopental sodium.

## 5. Conclusion

To conclude, we can say that the pharmacological study that we carried out on 4-phenyl-1,5-benzodiazepin-2-one and its long-chain derivatives **5a–d** having a structure similar to surfactants shows that all its products are slightly toxic. Therefore, **5a–d** products belong to category V, in the global system of classification of chemical substances.

Furthermore, we have shown that 4-phenyl-1,5-benzodiazepin-2-one and its derivatives do not possess catatonigenic and hypnotic actions at therapeutic doses of 100 and 200 mg/kg. 4-phenyl-1,5-benzodiazepin-2-one has no sedative effect of interest at the therapeutic doses studied. On the other hand, its derivatives have some.

In addition, at 100 mg/kg, all these products have the ability to prolong the hypnotic effect of thiopental sodium.

## Figures and Tables

**Table 1:** Spectral data ^1^H NMR, ^13^C NMR, and mass spectrometry.

Products	^1^H NMR spectrum (*δ* in ppm)	^13^C NMR spectrum (*δ* in ppm)	Mass spectrum MH^+^ (m/z)
4-Phenyl-1,5-benzodiazepine-2-one **3**	9.37 (s, NH); 8.35–7.15 (m, 9H); 3.66 (s, 2H)	167.80 (C = O); 158.64 (C = N); 139.91–137.62 (C aromatic); 130.98 (CH aromatic); 129.14–121.82 (C aromatic); 39.79 (CH_2_)	237
1-Octyl-4-phenyl-1,5-benzodiazepin-2-one **5a**	8.15–7.24 (m, 9H, CH); 4.35 (t, 2H, N-CH_2_); 3.55 (q, 2H, CH_2_-CO); 1.86–1.10 (m, 12H, CH_2_); 0.78 (t, 3H, CH_3_)	165.62 (C = O); 128.70–122.41 (C aromatic); 47.12 (CH_2_-N); 40.06 (CH_2_-CO); 14.03 (CH_3_)	349
1-Nonyl-4-phenyl-1,5-benzodiazepin-2-one **5b**	8.16–7.25 (m, 9H, CH); 4.30 (t, 2H, CH_2_-N); 3.55 (q, 2H, CH_2_-CO); 1.50–1.10 (m, 14H, CH_2_); 0.82 (t, 3H, CH_3_)	165.57 (C = O); 131.35–122.41 (C aromatic); 47.19 (CH_2_-N); 40.08 (CH_2_-CO); 14.09 (CH_3_)	363
1-Decyl-4-phenyl-1,5-benzodiazepin-2-one **5c**	8.17–7.26 (m, 9H, CH); 4.20 (t, 2H, CH_2_-N); 3.56 (q, 2H, CH_2_-CO); 1.50–1.10 (m, 16H, CH_2_); 0.84 (t, 3H, CH_3_)	165.51 (C = O); 128.75–122.41 (C aromatic); 47.22 (CH_2_-N); 40.12 (CH_2_-CO); 14.11 (CH_3_)	377
1-Dodecyl-4-phenyl-1,5-benzodiazepin-2-one **5d**	8.18–7.25 (m, 9H, CH); 4.96 (t, 2H, CH_2_-N); 3.58 (q, 2H, CH_2_-CO); 1.26–1.11 (m, 20H, CH_2_); 0.87 (t, 3H, CH_3_)	165.49 (s, CO); 162.18–122.40 (m, C aromatic); 47.26 (s, CH_2_-N); 40.14 (s, CH_2_-CO); 14.09 (s, CH_3_)	405

m = multiplet; q = quartet; s = singulet; t = triplet.

**Table 2:** Results of the sedative activity of 4-phenyl-1,5-benzodiazepin-2-one at doses of 100 and 200 mg/kg (*P* < 0.001 compared to controls).

		Witness	Reference (bromazepam 20 mg/kg)	4-Phenyl-1,5-benzodiazepin-2-one (100 mg/kg)	4-Phenyl-1,5-benzodiazepin-2-one (200 mg/kg)
Traction test	Percentage of falls (%)	0	100	0	0
	Average fall time (s)	0	10.0 ± 0.9	0	0
	Average recovery time (s)	0.50 ± 0.10	0	1.68 ± 0.10	6.12 ± 5.44
Chimney test	Average time to climb the chimney (s)	3.40 ± 0.50	>120	1.80 ± 1.64	8.16 ± 5.64
Hole-board test	Number of holes explored over 5 min	7 ± 1	0 ± 0	3.48 ± 2.22	3.36 ± 2.55
Rota-Rod test	Time spent on the stem (s)	120 ± 0	1.2 ± 0.90	120 ± 0	120 ± 0

**Table 3:** Results of the sedative activity of the products **5a–d** at doses of 100 and 200 mg/kg (*P* < 0.001 compared to controls).

		Witness	Reference (bromazepam 20 mg/kg)	**5a** (100 mg/kg)	**5a** (200 mg/Kg)	**5b** (100 mg/kg)	**5b** (200 mg/kg)	**5c** (100 mg/kg)	**5c** (200 mg/kg)	**5d** (100 mg/kg)	**5d** (200 mg/kg)
Traction test	Percentage of falls (%)	0	100	10	12	8	8	0	0	0	0
	Average fall time (s)	0	10.0 ± 0.9	8.52 ± 2.94	4.48 ± 0.87	25.33 ± 7.12	8.80 ± 2.22	0	0	0	0
	Average recovery time (s)	0.50 ± 0.10	0	2.63 ± 1.15	1.38 ± 0.39	4.93 ± 2.53	3.37 ± 2.15	9.02 ± 2.58	5.71 ± 0.21	17.34 ± 4.70	14.71 ± 3.75
Chimney test	Average time to climb the chimney (s)	3.40 ± 0.50	>120	12.14 ± 2.19	8.19 ± 3.50	23.78 ± 5.67	12.48 ± 3.04	38.80 ± 10.14	33.29 ± 5.67	87.40 ± 12.74	75.63 ± 5.19
Hole-board test	Number of holes explored over 5 min	7 ± 1	0 ± 0	0.88 ± 0.38	0.84 ± 0.43	1.28 ± 0.36	1.20 ± 0.41	1.72 ± 1.29	1.48 ± 0.86	2.00 ± 1.12	1.80 ± 1.05
Rota-Rod test	Time spent on stem (s)	120 ± 0	1.2 ± 0.90	16.50 ± 3.35	15.40 ± 9.83	23.2 ± 6.98	19.25 ± 6.60	35.50 ± 11.9	25.20 ± 8.98	64.08 ± 26.02	40.20 ± 18.70

**Table 4:** Results of the drug interaction of thiopental sodium with 4-phenyl-1,5-benzodiazepin-2-one (*P* < 0.001 compared to controls).

	Witness thiopental sodium (40 mg/kg)	Thiopental sodium (40 mg/kg) + 4-phenyl-1,5-benzodiazepin-2-one (100 mg/kg)
TE (s)	180 ± 60	143.40 ± 33.33
TS (min)	45 ± 20	140.60 ± 104.03

**Table 5:** Results of the drug interaction of thiopental sodium with **5i** (*P* < 0.001 compared to controls).

Time	Witness thiopental sodium (40 mg/kg)	Reference thiopental sodium (40 mg/kg) + bromazepam (30 mg/kg)	Thiopental sodium (40 mg/kg) + **5a** (100 mg/kg)	Thiopental sodium (40 mg/kg) + **5b** (100 mg/kg)	Thiopental sodium (40 mg/kg) + **5c** (100 mg/kg)	Thiopental sodium (40 mg/kg) + **5d** (100 mg/kg)
TE (s)	180 ± 60	105 ± 57	167 ± 50.49	99 ± 22.09	81 ± 29.98	117 ± 25
TS (min)	45 ± 20	231 ± 13.25	153 ± 59.61	118 ± 46.18	88 ± 25.31	82 ± 12.76
